# Do mobile applications foster sustainable mobility? Evidence from a field experiment

**DOI:** 10.1186/s41937-024-00129-y

**Published:** 2024-09-23

**Authors:** Alexander Goetz, Ioana Marinica, Harald Mayr, Luca Mosetti, Renate Schubert

**Affiliations:** 1 Swiss National Bank, Zurich, Switzerland; 2 ETH Zurich, Zurich, Switzerland; 3 University of Zurich, Zurich, Switzerland

**Keywords:** Sustainable mobility, Mobile application, Randomized controlled trial, C93, D12, Q50

## Abstract

Mobile applications hold promise to foster sustainable mobility behavior, but evaluations of their effectiveness are subject to a number of empirical challenges. We conduct a randomized controlled trial with three distinctive features: unobtrusive tracking of the control group, limited sample attrition, and a representative sample. In our study, 410 participants track their mobility behavior over a 5 week period. After 1 week, the treatment group engages with the user interface of the “Swiss Climate Challenge App”. The user interface combines information on individual $\hbox {CO}_2$ emissions with gamification features. We find a treatment effect that implies a $9.8\%$ reduction in emissions caused by access to the mobile application. While we lack the statistical power to exclude a zero average effect, we find statistically significant emission reductions in the second half of the intervention period, among subjects in medium population density areas, and among men. Our findings suggest that mobile applications could generate considerable net benefits, but larger studies will be needed for validation.

## Introduction

The transport sector poses a major challenge to the decarbonization of the global economy. In 2019, transportation was responsible for 24% of global $\hbox {CO}_2$ emissions from burning fossil fuels (International Energy Agency, 2020). Unless behavioral change, technology, and changes in the built environment can decouple transport emissions from economic activity and population growth, emissions in the transport sector will keep growing (Creutzig et al., 2014). Monetary incentives have been shown to reduce externalities associated with mobility (Hintermann et al., 2024; Tarduno, 2021; Kreindler, 2023) and to promote biking (Máca et al., 2020; Ciccone et al., 2021) as well as public transport use (Gravert and Olsson Collentine, 2021). Standard behavioral interventions like information provision, however, seem to be largely ineffective in this domain (Kristal and Whillans, 2020; Rosenfield et al., 2020; Hintermann et al., 2024).

Mobile applications offer new possibilities in the endeavor to foster sustainable mobility. A range of mobile applications in the spirit of “persuasive technology” (Fogg, 2002) are currently available (Froehlich et al., 2009; Jylhä et al., 2013; Jariyasunant et al., 2015; Cellina et al. 2019). Whether they indeed change mobility behavior is unclear. The meta-analysis of Sunio and Schmöcker (2017, p. 553) finds that *“methodologically robust studies are largely missing”*. Cellina et al. (2019) recently published the first randomized controlled trial on this question, but the authors point out three limitations of their study: obtrusive tracking, severe sample attrition, and a potentially unrepresentative sample.

In our study, we evaluate the “Swiss Climate Challenge App” (henceforth: SCC App) in a randomized controlled trial that addresses the aforementioned limitations. Study participants were recruited to participate in a study on mobility and to continuously use a tracking app for a period of 5 weeks. The tracking app comes with a plain user interface that shows only whether all necessary permissions are granted. After a 1-week pre-intervention period, the user interface of the treatment group switches to the SCC App for the rest of the study period. The SCC App provides graphical feedback on users’ personal mobility as well as additional features that rely on moral appeal, social comparison, and goal setting.^1^ The control group remains in the plain user interface, without any mention of sustainability aspects.

Our randomized controlled trial provides three main contributions. First, we utilize technology that allows unobtrusive tracking, limiting potential experimenter demand effects. In contrast to subjects in Cellina et al. (2019), participants in our study do not have to manually validate their trips. Second, we incentivize full study participation to limit sample attrition. A large share of the participation fee was conditional on compliance with tracking criteria and our final sample size of 410 compares favorably to the 52 reported by Cellina et al. (2019). Third, we use a representative population sample. Our study sample was recruited to represent the Swiss population in terms of age, gender, and language region.

Participants in the treatment group engage with the SCC App. $78\%$ of the treatment group open the SCC App at some point during the intervention period, $58\%$ open the SCC App on at least five different days, and $31\%$ use it on at least 14 different days (out of 28 days in the intervention period).

We use a standard difference-in-differences approach to evaluate the effect of the SCC App. Our results suggest that the SCC App reduces emissions by $9.8\%$, but this average effect is not statistically significant at conventional significance levels. We find substantial heterogeneity in this effect, with statistically significant reductions in emissions in the second half of the intervention period, in medium population density areas, and among male participants. This study is, to the best of our knowledge, the largest randomized controlled trial to evaluate the effects of mobile applications that aim to foster sustainable mobility behavior in the spirit of “persuasive technology”. Notwithstanding, larger studies will be needed to validate our results.

## Experimental design

We evaluate the SCC App in a randomized controlled trial. This section describes the functionalities of the SCC App, our recruiting procedure, and the study protocol.

The SCC App is a mobile application that automatically tracks the user’s mobility behavior with location and motion sensors. The underlying technology, provided by the company MotionTag, automatically detects the mode of transport with 92% accuracy (Molloy et al., 2020). It correctly detects more than 90% of airplane, car, subway, tram, and walk trips, but has some difficulty detecting bike, bus, train, and regional train trips (see Table 7 in Molloy et al., 2023) and may miss some trips due to gaps in GPS data (Mesaric et al., 2022). The SCC App uses the detected mode of transport in combination with the distance traveled to calculate the user’s $\hbox {CO}_2$ emissions.

**Fig. 1:**
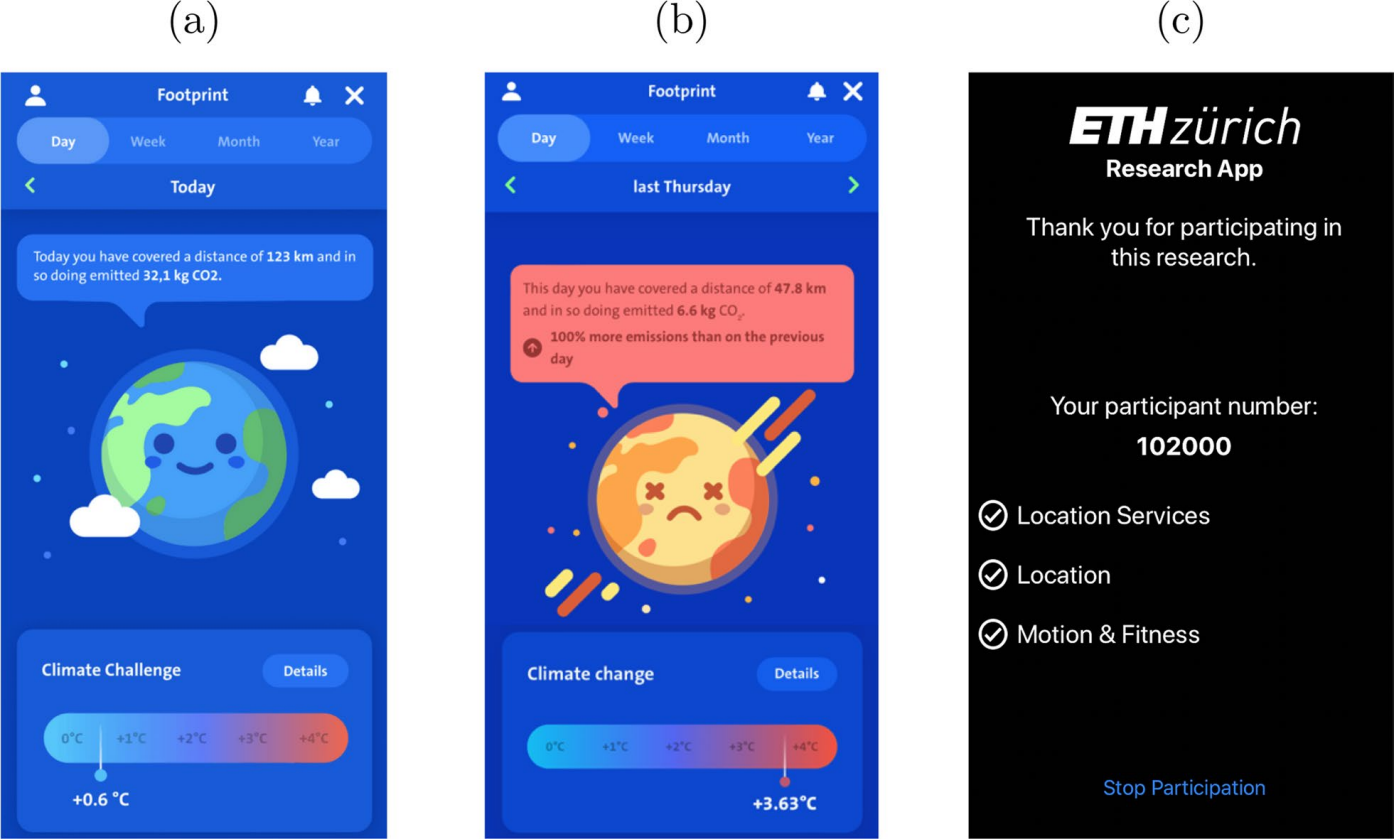
Home screens of the SCC App (**a**, **b**) and the ETH Research App (**c**)

The SCC App provides the user with graphical feedback on the environmental impact of her behavior and uses moral appeal, social comparison, and goal setting to motivate the user to reduce emissions. The home screen of the SCC App combines feedback with a moral appeal: it illustrates graphically how the user’s mobility behavior—if adopted by the entire world population—would affect global temperature. The respective temperature increase (e.g. $+0.6$  °C) is shown along with a happy-face earth for low values (see Fig. 1a) or a knocked-out earth for high values (see Fig. 1b). Users can also consult graphical feedback on the amount of CO_2_ they emit with their mobility behavior on any given day, week, or month, as well as a breakdown of their mobility behavior by means of transport and details for every trip they took. They can also look at a social comparison feature to compare their emissions with the regional (cantonal) average or with invited friends. In addition, users can accept personal challenges like “Take the bike every day of the week” and win symbolic badges.^2^

A market research company recruited the participants for this study. The company provided us with a representative sample of the Swiss population (based on gender, age, and language region) and was responsible for all communication with the participants. Participants were informed that they were going to take part in a study on mobility. To avoid experimenter demand effects, the notion of sustainability was not imparted to the control group until the end of the study period. Participants needed to have either an iOS or Android smartphone with internet access. They further had to be willing to allow us to track their location for the entire study duration. Participants received 10 Swiss Francs^3^ in exchange for taking part in the pre-intervention period of the study. In order to minimize sample attrition, participants received an additional 40 Swiss Francs if they complied with our instructions until the end of the intervention period. Both in the pre-intervention period and in the intervention period, remuneration was conditional on granting all necessary permissions for location tracking.

One thousand seven hundred and eleven study participants gave informed consent, filled out a survey at the beginning of the study, and successfully downloaded the mobile application. Among other variables, we collected data on the participants’ characteristics and various self-reported environmental behaviors. After filling out the survey, we provided the participants with the instructions for downloading the tracking app (described below) and for granting the necessary location tracking permissions on the mobile phone. The instructions also provided a number, with which the study participants could register in the tracking app.

We recruited participants from April 6, 2021 until April 18, 2021. After this onboarding period, participants tracked their mobility behavior in the pre-intervention period, which lasted from April 18 until April 26, 2021.^4^ Participants were instructed to use the “ETH Research App”, a specifically developed mobile application, to track their mobility behavior. This application uses the same location tracking technology as the SCC App, but it has a plain user interface that does not refer to environmental aspects of mobility. Users of the ETH Research App only see whether all necessary permissions for mobility tracking are granted, as illustrated in Fig. 1c. 1146 participants ($67\%$ of 1711) successfully used the ETH Research App during the entire pre-intervention period. Our budget constraint allowed us to invite 570 of these participants to take part in the intervention period. Before the start of the intervention period, we randomly assigned these 570 participants to the control and treatment group. Randomization was stratified on recruitment date and $\hbox {CO}_2$ emissions in the pre-intervention period. This randomization procedure ensured that participants in the control and treatment group had a comparable recruitment date and comparable emissions in the pre-intervention period.

In the 4-week intervention period, the control group continued to use the ETH Research App, while the treatment group was given access to the SCC App. On April 27, participants in the treatment group received an email encouraging them to use the SCC App with its various features. They only needed to tap a button in the ETH Research App, which then turned into the full SCC App. At the same time, participants in the control group received an email which asked them to continue tracking with the ETH Research App for the rest of the study period.^5^ 410 ($72\%$ of 570) participants successfully tracked their mobility behavior in the intervention period. We observe no statistically significant difference in tracking success between the control and treatment group.^6^ Finally, participants were asked to fill out a final survey.

To summarize, $67\%$ of 1711 participants completed the pre-intervention period and $72\%$ of 570 participants invited for the intervention period completed the intervention period. The resulting sample consists of 410 participants.^7^

## Data and descriptive statistics

We have detailed information about each trip taken by each user. For the purpose of this study, we use data on the date of a trip, the mode of transport, and the associated $\hbox {CO}_2$ emissions.^8^

Table 1 reports the mobility behavior of our sample during the pre-intervention period as well as participants’ characteristics. Column (1) shows the means and standard deviations (in parentheses) of the sample. Columns (2) and (3) present the same statistics for the control and treatment group. Column (4) reports whether the differences between control and treatment group are statistically significant (*p*-values). According to column (1), participants emit on average 6.3 kg of $\hbox {CO}_2$ per day during the pre-intervention period. The participants take an average of 6.9 trips (44.2 km). 2.1 of these trips (32%, 30.2 km) are by car, 0.9 (12%, 10.3 km) by public transport, 0.4 (6%, 1.4 km) by bike, and 3.4 (51%, 2.1 km) on foot. Compared to the 2021 Mobility and Transport Microcensus (Bundesamt für Statistik, 2023), our sample has similar modal shares, but it is more mobile (both in terms of trips and distance). A potential explanation for this difference could be increased mobility following the partial lifting of COVID-19 restrictions in the pre-intervention period. Participants in the Mobility and Transport Microcensus take an average of 3.8 trips (30 km) across modes, 1.4 of those (37%, 20.8 km) by car, 0.5 (13%, 5.9 km) by public transport, 0.2 (5%, 0.7 km) by bike, and 1.6 (42%, 1.6 km) on foot. Study participants were recruited to be representative of the Swiss population in terms of age, gender, and language region. Our sample is on average 44 years old, has a similar number of women and men, and a German speaking majority. Column (4) indicates that the differences between the treatment group and the control group are statistically insignificant.^9^ Hence, Table 1 suggests that the randomization procedure achieved a balance on observable characteristics.

**Table 1:** Pre-intervention data

Variable	(1)	(2)	(3)	(4)
	Total	Control	Treatment	*p*-value
CO_2_ emissions (kg)	6.31	5.94	6.66	0.31
	(50.48)	(46.46)	(53.96)	
Trips:				
Total trips	6.86	6.76	6.96	0.61
	(28.45)	(27.90)	(29.01)	
Car	2.20	2.16	2.23	0.75
	(13.40)	(13.69)	(13.16)	
Public transport	0.79	0.76	0.82	0.63
	(8.82)	(7.99)	(9.53)	
Bike	0.39	0.38	0.41	0.57
	(4.57)	(4.36)	(4.76)	
Walking	3.48	3.46	3.50	0.85
	(17.23)	(16.70)	(17.76)	
Modal share (trips):				1.00
Car	0.32	0.32	0.32	
Public transport	0.12	0.11	0.12	
Bike	0.06	0.06	0.06	
Walking	0.51	0.51	0.50	
Distance traveled:				
Total distance	44.22	40.26	47.92	0.09
	(325.96)	(253.31)	(380.34)	
Car	30.16	28.64	31.57	0.41
	(252.76)	(235.84)	(267.77)	
Public transport	10.31	7.92	12.54	0.10
	(205.78)	(114.98)	(263.13)	
Bike	1.43	1.57	1.30	0.45
	(24.85)	(30.33)	(18.36)	
Walking	2.08	2.13	2.04	0.64
	(13.69)	(15.07)	(12.29)	
Gender:				0.53
Male	0.53	0.55	0.51	
Female	0.47	0.45	0.49	
Main language:				0.19
German	0.64	0.63	0.66	
French	0.30	0.33	0.27	
Italian	0.06	0.04	0.07	
Age	43.96	44.07	43.86	0.89
	(15.22)	(15.28)	(15.20)	
Observations	410	198	212	

The table depicts daily mobility behavior during the 1-week pre-intervention period and participants’ characteristics. Column (1) depicts means for the estimation sample (with standard errors of numerical variables in parentheses). Column (2) and column (3) depict the same data for the control and treatment group, respectively. Column (4) depicts *p*-values for the differences between control and treatment group. The corresponding *p*-values are obtained from a t-test for numerical variables, and from a chi square test for the categorical variables (modal share, gender and main language)

We further analyze data on interactions with the SCC App. So-called screen events are recorded every time a user views the app’s user interface. An event is annotated with the timestamp and the page that was viewed. These data allow us to gain insights into participants’ engagement with the SCC App and to identify the most frequently used features.

**Fig. 2:**
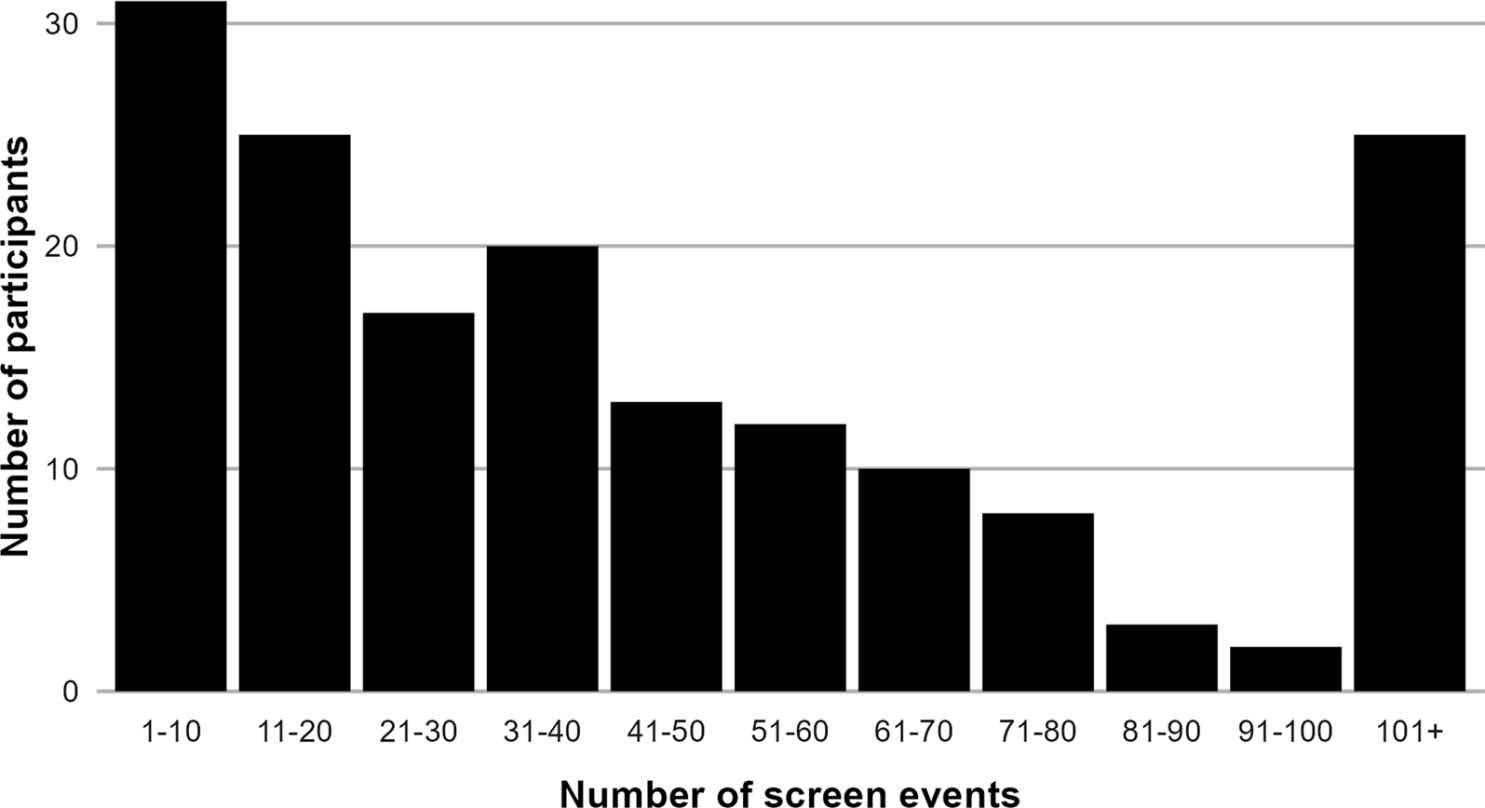
Histogram of screen events by participant

The majority of participants in the treatment group uses the SCC App repeatedly. 166 of the 212 participants in the treatment group ($78\%$) use the SCC App at some point during the intervention period. Figure 2 shows a histogram of the total number of SCC App screen events (i.e. interactions with the user interface) among these participants. 31 participants use the SCC App sporadically, with up to 10 screen events, but many use it more extensively. 25 participants have more than 100 SCC App screen events. Most of these screen events ($53\%$) pertain to the SCC App home screen as shown in Fig. 1a and b, but users also view the social comparisons ($8\%$), individual trips ($7\%$), mobility challenges ($6\%$), among other pages.

**Fig. 3:**
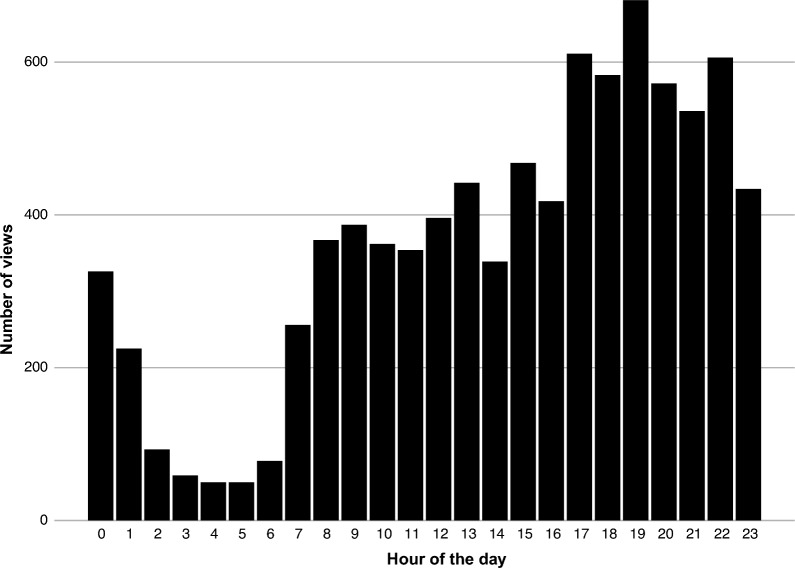
Screen events by hour of the day

**Fig. 4:**
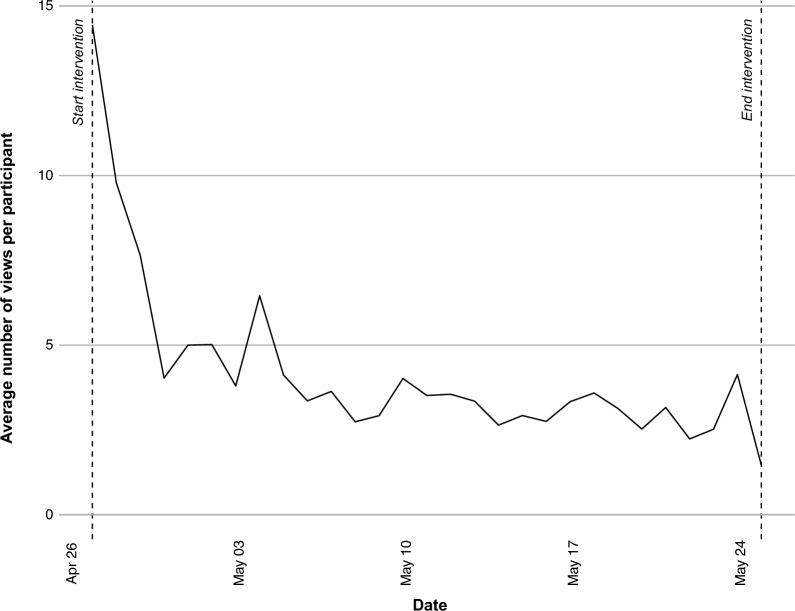
Screen events by date

We further analyze the distribution of screen events by hour of the day and over the course of the intervention period. Figure 3 shows that participants in the treatment group use the SCC App throughout the day, but most screen events occur in the evening. This pattern is consistent with the idea that participants use the SCC App to reflect on their mobility behavior at the end of the day. Figure 4 shows that the number of screen events is very high at the beginning of the intervention period. After a few days, the number of screen events stabilizes at around 4 per participant and day. This pattern suggests that participants use the SCC App intensively at the beginning of the intervention period, but then use it more sporadically.

## Estimation and results

We use a difference-in-differences regression framework to estimate the effect of the SCC App on the the log of $\hbox {CO}_2$ emissions. To deal with zero values, we calculate $log(CO_{2i,t}+1)$,^10^ where *i* denotes individual participants and *t* denotes time periods (i.e. pre-intervention period or intervention period):

1 $$\begin{aligned} log(CO2_{i,t}+1) =\, \alpha + \beta Treatment_i + \gamma Time_{t} + \delta (Treatment_i\times Time_t) + \phi X_i + \epsilon _{i,t} \end{aligned}$$

We regress $log(CO2_{i,t}+1)$ on a treatment dummy variable ($Treatment_i$ is equal to 1 if participant *i* is allocated to the treatment group, 0 otherwise), a dummy variable for observations after the start of the intervention ($Time_t$ is equal to 1 if the observation is from the intervention period, 0 otherwise), and the interaction of both dummy variables ($Treatment_i\times Time_t$). The coefficient for the latter, $\delta$, is our parameter of interest, as it measures the effect of the SCC App on $\hbox {CO}_2$ emissions. We estimate Eq. (1) with OLS and calculate heteroskedasticity robust standard errors. Our basic specification does not include additional control variables $X_i$. In addition, we estimate specifications controlling for demographic characteristics (age, gender, income, education, and language), location (population density and distance to public transport), and pre-intervention mobility behavior ($\hbox {CO}_2$ emissions, total trips, car trips, public transport trips, bike trips, and walk trips).

Table 2 shows the results of our analysis. Column (1) shows the OLS regression coefficient (and standard error in parenthesis) for the specification that regresses $log({{CO}_2}+1)$ on *Treatment*, *Time*, and $Treatment~\times ~Time$. We find no statistically significant coefficient for *Treatment*. The coefficient for *Time* indicates that emissions are $28\%$ higher during the intervention period compared to the pre-intervention period.^11^ This time pattern may be related to the partial lifting of COVID-19 regulations during the pre-intervention period. Our coefficient of interest for the interaction term $Treatment \times Time$ implies a $9.8\%$ reduction in emissions, but this effect is statistically insignificant at conventional significance levels ($p>0.10$).

**Table 2:** Regression results

	$$log({{CO}_2}+1)$$			
	(1)	(2)	(3)	(4)
Time	$$0.247^{**}$$	$$0.247^{**}$$	$$0.247^{**}$$	$$0.247^{***}$$
	(0.118)	(0.116)	(0.111)	(0.060)
Treatment	0.157	0.162	$0.200^{*}$	$0.042^{*}$
	(0.123)	(0.122)	(0.115)	(0.025)
Treatment × Time	$-0.103$	$-0.103$	$-0.103$	$-0.103$
	(0.163)	(0.161)	(0.153)	(0.076)
Age		$-0.005^{*}$	$-0.004^{*}$	$-0.002$
		(0.003)	(0.002)	(0.001)
Female (reference: male)		$$-0.257^{***}$$	$$-0.281^{***}$$	$$-0.051$$
		(0.084)	(0.080)	(0.040)
Monthly income CHF 9000 and higher		0.060	0.029	0.054
		(0.081)	(0.078)	(0.039)
Higher education		$$0.302^{***}$$	$$0.252^{***}$$	0.029
		(0.085)	(0.081)	(0.040)
French (reference: German)		$-0.146$	$-0.159^{*}$	$-0.043$
		(0.096)	(0.090)	(0.044)
Italian (reference: German)		0.015	$-0.056$	$-0.051$
		(0.169)	(0.164)	(0.075)
Medium density zip code (reference: high)			$$0.697^{***}$$	$$0.106^{**}$$
			(0.097)	(0.051)
Low density zip code (reference: high)			$$0.967^{***}$$	$$0.094^{*}$$
			(0.106)	(0.056)
Distance to public transport			0.000	$-0.001$
			(0.002)	(0.001)
Log pre-intervention CO_2_ emissions				$$0.766^{***}$$
				(0.033)
Pre-intervention trips (total)				$-0.001$
				(0.002)
Pre-intervention trips by car				$$0.008^{***}$$
				(0.003)
Pre-intervention trips by public transport				0.003
				(0.004)
Pre-intervention trips by bike				0.003
				(0.005)
Constant	$$3.139^{***}$$	$$3.393^{***}$$	$$2.866^{***}$$	$$0.680^{***}$$
	(0.089)	(0.175)	(0.175)	(0.126)
Observations	820	820	820	820

The table presents estimates for the difference-in-differences model in Eq. (1). Column (1) depicts a regression of $log({{CO}_2}+1)$ on $Treatment, Time$, and $Treatment \times Time$. Column (2) adds participants’ age, gender language, above-median income, and higher education as additional control variables. Column (3) adds degree of urbanisation and distance to public transport (in km) as control variables. Column (4) further adds pre-intervention mobility behavior as control variables

The values in parentheses represent heteroskedasticity robust standard errors

*, **, ***, indicate statistical significance at the 10%, 5%, and 1% level

The specification in column (2) of Table 2 includes control variables for age, gender, income, education, and language. Compared to the specification in column (1), the coefficient of interest in column (2) is equal in size but remains statistically insignificant. The standard error in the second specification remains also equal in size, indicating that the set of control variables used for this specification cannot increase the precision of our main estimate. The specification in column (3) of Table 2 includes additional control variables for polulation density and the distance to public transport. The coefficient of interest in this specification is equal in size to the coefficients in columns (1) and (2). The standard error is only slightly lower than in the first two specifications.

The specification in column (4) of Table 2 further includes pre-intervention mobility variables. Although this specification does not change the coefficient of interest, it increases its precision compared to the specifications in columns (1), (2), and (3). Nevertheless, the coefficient remains statistically insignificant. The most important control variables appear to be pre-intervention emissions, pre-intervention car trips, and population density.

The results in Table 2 are not susceptible to outliers. Table 3 in Appendix 4 shows very similar results for the sample that excludes observations in the top $1\%$ of the outcome variable.

The effect of the SCC App may vary over time, as Fig. 4 suggests decreasing engagement over time. We test for this possibility by estimating Eq. (1) separately for the first and second half of the intervention period. Tables 4 and 5 in Appendix 4 show that the coefficient of interest is small and statistically insignificant in the first half of the intervention period, but larger and statistically significant ($p<0.10$) in the second half of the intervention period. This finding suggests that, despite decreasing engagement, the effect of the SCC App may increase over time.

Tables 6,  7, 8 and 9 in Appendix 4 show results for individual modes of transport. All coefficients of interest in these tables are statistically insignificant. If taken at face value, these coefficients suggest reduced car use (Table 6), limited effects on public transport (Table 7) and bike use (Table 8), and more trips on foot (Table 9). These tables yield other notable findings. Table 6 shows that the car is used more by high-income participants in medium and low density zip codes, who have more emissions, more car trips, and fewer public transport trips in the pre-intervention period. Table 7 shows that public transport is negatively associated with age and French speakers. Table 8 shows that bike use is associated with men, income, and high pre-intervention period emissions. In Table 9, walking is negatively associated with distance to public transport, emissions, car trips, public transport trips, and bike trips in the pre-intervention period.

Tables 10, 11, 12, 13, 14, 15, 16, 17, 18, 19 and 20 in Appendix 4 show results for $\hbox {CO}_2$ emissions among different sub-samples. We split our sample by low/medium/high population density (Tables 10, 11 and 12), below/above median emissions in the pre-intervention period (Tables 13 and 14), below/above median age (Tables 15 and 16), male/female gender (Tables 17 and 18), and sub-samples with only those treatment participants who did not/did interact with the SCC App (Tables 19 and 20). These sub-sample regressions reveal two interesting dimensions of effect heterogeneity: population density and gender. We find an *increase* in $\hbox {CO}_2$ emissions in low density areas (specification 4 in Table 10, $p<0.10$), but a decrease in medium density areas (specification 4 in Table 11, $p<0.10$) and high density areas (Table 12, not statistically significant). This finding suggests that the SCC App may be more effective where public transport is more accessible, and potentially counterproductive in areas where car use is more prevalent. Such counteracting effects, sometimes referred to as “boomerang effects” (Schultz et al., 2007), have been found in related research on mobility behavior (Gessner et al., 2024). The coefficient of interest is also statistically significant in the male sub-sample (specification 4 in Table 17, $p<0.05$). This finding is consistent with Hintermann et al. (2024), who find a particularly strong effect of a mobility pricing intervention among men.

We also test whether the SCC App has an effect on environmental behaviors other than the target behavior (Tiefenbeck et al., 2013; Jessoe et al., 2021; Goetz et al., 2024). Two surveys at the beginning and end of our study provide data on self reported environmental behaviors. We do not find evidence for spillover effects. Appendix 5 depicts the details of this analysis.

On average, we do not find statistically significant effects of the SCC App on mobility behavior.^12^ However, we find substantial heterogeneity in the effect of the SCC App, with reductions in emissions in the second half of the intervention period, in medium population density areas, and among male participants.

## Conclusions

We evaluate the effect of mobile applications on sustainable mobility. Our randomized controlled trial provides three methodological contributions: unobtrusive tracking of the control group, limited sample attrition, and a representative population sample. We find an effect on mobility-related $\hbox {CO}_2$ emissions that is of substantial magnitude, but not statistically significant. COVID-19 may well have contributed to the lack of statistical significance. The lifting of restrictions likely impacted participants very differently, and some participants may have suffered from COVID-19 during our study period. The resulting variation in mobility behavior arguably makes it harder to detect the effect of the SCC App.

The large magnitude of our effect estimate suggests that the SCC App improves welfare by reducing $\hbox {CO}_2$ emissions. The $9.8\%$ reduction in emissions compares favorably to similar interventions in the literature. As an example, Hintermann et al. (2024) use similar technology in the Swiss context and find a $4\%$ reduction caused by an intervention that includes not only information, but also mobility pricing. Our large effect estimate could indicate that the SCC App was particularly successful in motivating behavior change. This would imply substantial benefits through reduced environmental damage and associated social costs. A $9.8\%$ reduction of yearly mobility emissions of 1.21 $\hbox {tCO}_2$ (Bundesamt für Umwelt, 2024) corresponds to 0.12 $\hbox {tCO}_2$ per person and year. Valued at the social cost of carbon of CHF 185 (Rennert et al., 2022), this reduction is worth CHF 22. The marginal costs of digital applications like the SCC App are typically close to zero. If development costs are considered sunk, the welfare effect of the SCC App is likely positive. Given the statistically insignificant estimate, we caution the reader to take this interpretation with a grain of salt.

Further research on behavioral mobility interventions is imperative—not least because alternative interventions like bans or price increases for $\hbox {CO}_2$-emitting mobility suffer from strong acceptability problems. Interventions based on mobile apps may have a substantially higher degree of acceptability. New evaluation approaches may be necessary to detect potentially small effects of behavioral mobility interventions. We see particular promise in the use of anonymized mobile network data, which may allow researchers to dispense with paid participant tracking and conduct unobtrusive studies at scale. Offering a mobile application like the SCC App to a group that can be identified in mobile network data, treatment effects could be credibly estimated in a difference-in-differences approach. We also see potential in further investigating treatment effect heterogeneity. Our results suggest that mobility interventions could be particularly effective if targeted at specific population groups or areas. Future research may scrutinize the strong effects on men and participants in medium density areas, and develop interventions that are tailored to the needs of specific population groups.

## Figures and Tables

**Fig. 5:**
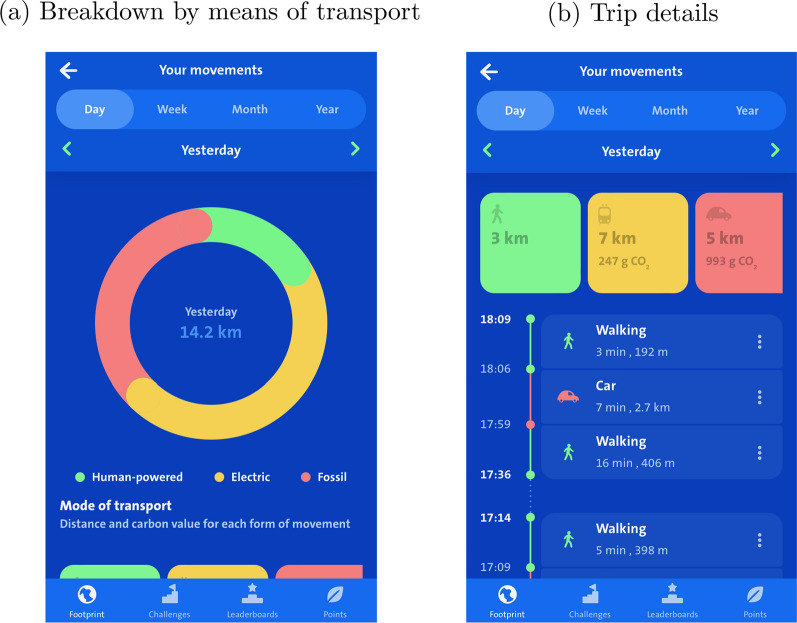
Graphical feedback on mobility behavior

**Fig. 6:**
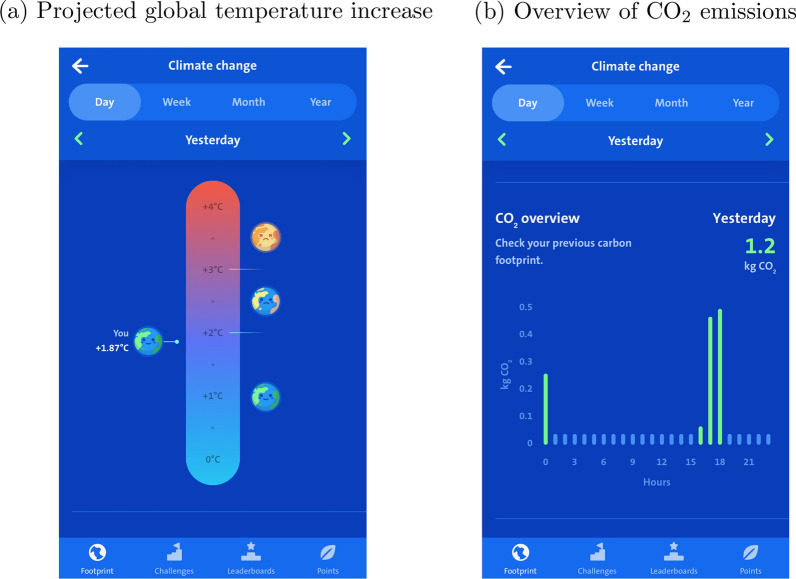
Moral appeal features

**Fig. 7:**
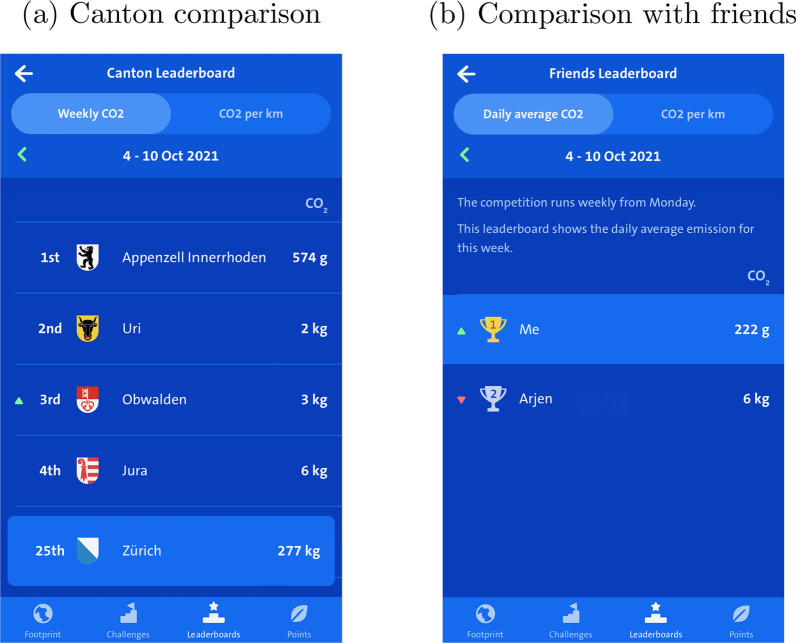
Social comparison features

**Fig. 8:**
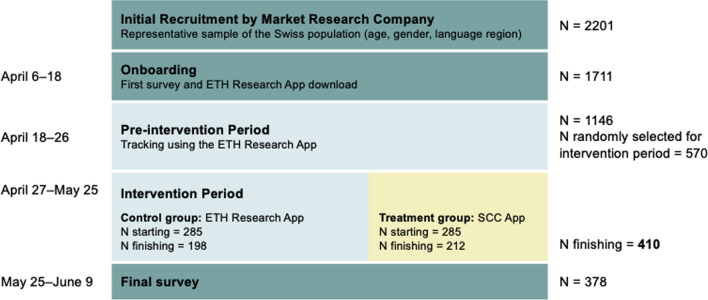
Study schedule

## Data Availability

The datasets used and/or analysed during the current study are available from the corresponding author on reasonable request.

## Transcriptions of intervention emails

**Treatment group intervention email**

Dear Study Participant,

Thank you for participating in this ETH study on mobility.

As of today, the ETH Research App on your device has an additional feature. By tapping on “Open Climate Challenge” at the bottom of the App’s home screen, you can open the Swiss Climate Challenge App.

The Swiss Climate Challenge App allows you to observe how much $\hbox {CO}_2$ you produce with your mobility, the breakdown of your mobility behavior by means of transport, and what kind of impact this has on the environment. You can also take part in various in-app challenges. We encourage you to interact with these various functionalities.

If you want, you can also invite your friends to download the Swiss Climate Challenge App from the App Store or Play Store, and use the App’s comparison feature. Please note that you are under no obligation to activate this feature.

The location tracking procedure and privacy policy remain the same. You do not need to make any further changes to the settings on your device.

You have already earned 10 Swiss Francs. You can earn another 40 Swiss Francs if you continue to participate in the study until May 24th. We would like to remind you that this additional remuneration is conditional upon granting the app all of the required location tracking permissions at all times.

Thank you for your commitment to this study!

Best regards,

The study team

**Control group intervention email**

Dear Study Participant,

Thank you for participating in this ETH study on mobility.

You have already earned 10 Swiss Francs. You can earn another 40 Swiss Francs if you continue to participate in the study until May 24th. We would like to remind you that this additional remuneration is conditional upon granting the app all of the required location tracking permissions at all times.

Thank you for your commitment to this study!

Best regards,

The study team

## Study schedule

See Fig. 8.

## Additional tables

See Tables 3, 4, 5, 6, 7, 8, 9, 10, 11, 12, 13, 14, 15, 16, 17, 18, 19 and 20.

**Table 3:** Regression results (outliers excluded)

	$$log({CO_{2}}+1)$$			
	(1)	(2)	(3)	(4)
Time	$$0.247^{**}$$	$$0.247^{**}$$	$$0.247^{**}$$	$$0.247^{***}$$
	(0.116)	(0.115)	(0.108)	(0.060)
Treatment	0.135	0.138	0.176	0.041
	(0.121)	(0.120)	(0.113)	(0.026)
Treatment × Time	$-0.112$	$-0.112$	$-0.112$	$-0.112$
	(0.160)	(0.158)	(0.149)	(0.076)
Age		$-0.004$	$-0.004$	$-0.001$
		(0.003)	(0.002)	(0.001)
Female (reference: male)		$$-0.224^{***}$$	$$-0.251^{***}$$	$$-0.045$$
		(0.083)	(0.078)	(0.040)
Monthly income CHF 9000 and higher		0.072	0.037	0.048
		(0.080)	(0.076)	(0.039)
Higher education		$$0.322^{***}$$	$$0.274^{***}$$	0.041
		(0.084)	(0.080)	(0.040)
French (reference: German)		$$-0.168^{*}$$	$$-0.188^{**}$$	$$-0.046$$
		(0.094)	(0.087)	(0.045)
Italian (reference: German)		0.046	$-0.015$	$-0.049$
		(0.170)	(0.163)	(0.076)
Medium density zip code (reference: high)			$$0.685^{***}$$	$$0.115^{**}$$
			(0.094)	(0.051)
Low density zip code (reference: high)			$$1.015^{***}$$	$$0.117^{**}$$
			(0.105)	(0.056)
Distance to public transport			0.000	$-0.001$
			(0.002)	(0.001)
Log pre-intervention CO_2_ emissions				$$0.747^{***}$$
				(0.033)
Pre-intervention trips (total)				$-0.001$
				(0.002)
Pre-intervention trips by car				$$0.009^{***}$$
				(0.003)
Pre-intervention trips by public transport				0.003
				(0.004)
Pre-intervention trips by bike				0.004
				(0.005)
Constant	$$3.114^{***}$$	$$3.306^{***}$$	$$2.782^{***}$$	$$0.693^{***}$$
	(0.089)	(0.170)	(0.169)	(0.128)
Observations	806	806	806	806

The table presents estimates for the difference-in-differences model in Eq. (1) excluding observations in the top $1\%$ of $log({CO_{2}}+1)$. Column (1) depicts a regression of $log({CO_{2}}+1)$ on $Treatment, Time$, and $Treatment \times Time$. Column (2) adds participants’ age, gender language, above-median income, and higher education as additional control variables. Column (3) adds degree of urbanisation and distance to public transport (in km) as control variables. Column (4) further adds pre-intervention mobility behavior as control variables

The values in parentheses represent heteroskedasticity robust standard errors *, **, *** indicate statistical significance at the 10%, 5%, and 1% level

**Table 4:** Regression results (first half of intervention period)

	$$log({CO_{2}}+1)$$			
	(1)	(2)	(3)	(4)
Time (first half of intervention period)	0.108	0.108	0.108	$0.108^{*}$
	(0.123)	(0.121)	(0.114)	(0.061)
Treatment	0.157	0.162	$0.203^{*}$	$0.041^{*}$
	(0.123)	(0.122)	(0.115)	(0.023)
Treatment × Time (first half of intervention period)	$-0.032$	$-0.032$	$-0.032$	$-0.032$
	(0.168)	(0.166)	(0.157)	(0.079)
Age		$-0.004$	$-0.004$	$-0.001$
		(0.003)	(0.003)	(0.001)
Female (reference: male)		$$-0.263^{***}$$	$$-0.288^{***}$$	$$-0.050$$
		(0.087)	(0.082)	(0.041)
Monthly income CHF 9000 and higher		0.079	0.045	$0.071^{*}$
		(0.084)	(0.079)	(0.040)
Higher education		$$0.333^{***}$$	$$0.280^{***}$$	0.057
		(0.087)	(0.084)	(0.044)
French (reference: German)		$$-0.176^{*}$$	$$-0.190^{**}$$	$$-0.070$$
		(0.100)	(0.092)	(0.046)
Italian (reference: German)		$-0.004$	$-0.079$	$-0.075$
		(0.176)	(0.170)	(0.089)
Medium density zip code (reference: high)			$$0.742^{***}$$	$$0.141^{***}$$
			(0.098)	(0.052)
Low density zip code (reference: high)			$$1.023^{***}$$	$$0.131^{**}$$
			(0.110)	(0.063)
Distance to public transport			0.000	$-0.001$
			(0.002)	(0.001)
Log pre-intervention CO_2_ emissions				$$0.775^{***}$$
				(0.032)
Pre-intervention trips (total)				$-0.002$
				(0.002)
Pre-intervention trips by car				$$0.010^{***}$$
				(0.003)
Pre-intervention trips by public transport				0.006
				(0.004)
Pre-intervention trips by bike				0.002
				(0.006)
Constant	$$3.139^{***}$$	$$3.370^{***}$$	$$2.807^{***}$$	$$0.578^{***}$$
	(0.089)	(0.177)	(0.177)	(0.126)
Observations	820	820	820	820

The table presents estimates for the difference-in-differences model in Eq. (1), with the intervention period restricted to the first 2 weeks. Column (1) depicts a regression of $log({CO_{2}}+1)$ on $Treatment, Time$, and $Treatment \times Time$. Column (2) adds participants’ age, gender language, above-median income, and higher education as additional control variables. Column (3) adds degree of urbanisation and distance to public transport (in km) as control variables. Column (4) further adds pre-intervention mobility behavior as control variables

The values in parentheses represent heteroskedasticity robust standard errors. *, **, ***, indicate statistical significance at the 10%, 5%, and 1% level

**Table 5:** Regression results (second half of intervention period)

	$$log({CO_{2}}+1)$$			
	(1)	(2)	(3)	(4)
Time (second half of intervention period)	$$0.227^{*}$$	$$0.227^{*}$$	$$0.227^{**}$$	$$0.227^{***}$$
	(0.121)	(0.120)	(0.114)	(0.066)
Treatment	0.157	0.163	$0.201^{*}$	$0.041^{*}$
	(0.123)	(0.122)	(0.115)	(0.025)
Treatment × Time (second half of intervention period)	$-0.154$	$-0.154$	$-0.154$	$-0.154^{*}$
	(0.168)	(0.166)	(0.158)	(0.086)
Age		$-0.005^{*}$	$-0.005^{*}$	$-0.002$
		(0.003)	(0.003)	(0.001)
Female (reference: male)		$$-0.238^{***}$$	$$-0.263^{***}$$	$$-0.034$$
		(0.087)	(0.083)	(0.045)
Monthly income CHF 9000 and higher		0.052	0.021	0.046
		(0.084)	(0.081)	(0.044)
Higher education		$$0.282^{***}$$	$$0.231^{***}$$	0.001
		(0.088)	(0.085)	(0.045)
French (reference: German)		$-0.117$	$-0.129$	$-0.013$
		(0.099)	(0.092)	(0.049)
Italian (reference: German)		0.007	$-0.065$	$-0.064$
		(0.187)	(0.183)	(0.089)
Medium density zip code (reference: high)			$$0.705^{***}$$	$$0.105^{*}$$
			(0.101)	(0.059)
Low density zip code (reference: high)			$$0.973^{***}$$	0.089
			(0.110)	(0.061)
Distance to public transport			0.000	$-0.001$
			(0.002)	(0.001)
log pre-intervention CO_2_ emissions				$$0.771^{***}$$
				(0.035)
Pre-intervention trips (total)				$-0.002$
				(0.002)
Pre-intervention trips by car				$$0.009^{***}$$
				(0.003)
Pre-intervention trips by public transport				0.002
				(0.005)
Pre-intervention trips by bike				0.006
				(0.005)
Constant	$$3.139^{***}$$	$$3.390^{***}$$	$$2.859^{***}$$	$$0.673^{***}$$
	(0.089)	(0.179)	(0.181)	(0.133)
Observations	820	820	820	820

$^{1}$ The table presents estimates for the difference-in-differences model in Eq. (1), with the intervention period restricted to the last 2 weeks. Column (1) depicts a regression of $log({CO_{2}}+1)$ on $Treatment, Time$, and $Treatment \times Time$. Column (2) adds participants’ age, gender language, above-median income, and higher education as additional control variables. Column (3) adds degree of urbanisation and distance to public transport (in km) as control variables. Column (4) further adds pre-intervention mobility behavior as control variables

The values in parentheses represent heteroskedasticity robust standard errors. *, **, ***, indicate statistical significance at the 10%, 5%, and 1% level

**Table 6:** Regression results (car trips)

	$$log(Distance+1)$$			
	(1)	(2)	(3)	(4)
Time	$$0.391^{**}$$	$$0.391^{**}$$	$$0.391^{***}$$	$$0.391^{***}$$
	(0.158)	(0.155)	(0.146)	(0.079)
Treatment	0.212	0.220	$0.280^{*}$	0.072
	(0.172)	(0.171)	(0.159)	(0.058)
Treatment × Time	$-0.168$	$-0.168$	$-0.168$	$-0.168$
	(0.218)	(0.215)	(0.201)	(0.105)
Age		$-0.005$	$-0.005$	$-0.002$
		(0.004)	(0.003)	(0.002)
Female (reference: male)		$$-0.301^{***}$$	$$-0.341^{***}$$	$$-0.081$$
		(0.113)	(0.106)	(0.054)
Monthly income CHF 9000 and higher		0.166	0.121	$$0.139^{***}$$
		(0.109)	(0.103)	(0.053)
Higher education		$$0.447^{***}$$	$$0.372^{***}$$	0.019
		(0.115)	(0.110)	(0.058)
French (reference: German)		$-0.145$	$-0.158$	$-0.033$
		(0.128)	(0.118)	(0.058)
Italian (reference: German)		0.179	0.058	0.007
		(0.211)	(0.210)	(0.100)
Medium density zip code (reference: high)			$$1.070^{***}$$	$$0.217^{***}$$
			(0.130)	(0.071)
Low density zip code (reference: high)			$$1.390^{***}$$	$$0.170^{**}$$
			(0.145)	(0.084)
Distance to public transport			$-0.000$	$-0.002$
			(0.002)	(0.002)
Log pre-intervention CO_2_ emissions				$$1.022^{***}$$
				(0.041)
Pre-intervention trips (total)				$-0.003$
				(0.002)
Pre-intervention trips by car				$$0.011^{***}$$
				(0.004)
Pre-intervention trips by public transport				$$-0.014^{**}$$
				(0.006)
Pre-intervention trips by bike				0.011
				(0.007)
Constant	$$4.370^{***}$$	$$4.562^{***}$$	$$3.782^{***}$$	$$1.174^{***}$$
	(0.126)	(0.237)	(0.233)	(0.181)
Observations	820	820	820	820

The table presents estimates for the difference-in-differences model in Eq. (1), with the weekly distance of car trips as outcome variable. Column (1) depicts a regression of $log(Distance+1)$ on $Treatment, Time$, and $Treatment \times Time$. Column (2) adds participants’ age, gender language, above-median income, and higher education as additional control variables. Column (3) adds degree of urbanisation and distance to public transport (in km) as control variables. Column (4) further adds pre-intervention mobility behavior as control variables

The values in parentheses represent heteroskedasticity robust standard errors. *, **, ***, indicate statistical significance at the 10%, 5%, and 1% level

**Table 7:** Regression results (public transport trips)

	$$log(Distance+1)$$			
	(1)	(2)	(3)	(4)
Time	$$0.358^{*}$$	$$0.358^{*}$$	$$0.358^{**}$$	$$0.358^{***}$$
	(0.193)	(0.185)	(0.181)	(0.137)
Treatment	0.175	0.155	0.130	0.126
	(0.204)	(0.197)	(0.193)	(0.135)
Treatment × Time	$-0.028$	$-0.028$	$-0.028$	$-0.028$
	(0.274)	(0.264)	(0.259)	(0.194)
Age		$$-0.020^{***}$$	$$-0.020^{***}$$	$$-0.013^{***}$$
		(0.004)	(0.004)	(0.003)
Female (reference: male)		$$-0.393^{***}$$	$$-0.371^{***}$$	$$-0.154$$
		(0.138)	(0.134)	(0.103)
Monthly income CHF 9000 and higher		$-0.107$	$-0.077$	0.043
		(0.134)	(0.133)	(0.102)
Higher education		$-0.286^{*}$	$-0.241^{*}$	0.131
		(0.148)	(0.146)	(0.114)
French (reference: German)		$$-0.821^{***}$$	$$-0.793^{***}$$	$$-0.496^{***}$$
		(0.155)	(0.152)	(0.121)
Italian (reference: German)		$$-1.017^{***}$$	$$-0.991^{***}$$	$$-0.403^{*}$$
		(0.285)	(0.290)	(0.238)
Medium density zip code (reference: high)			$$-0.562^{***}$$	0.140
			(0.154)	(0.121)
Low density zip code (reference: high)			$$-1.072^{***}$$	$$-0.349^{**}$$
			(0.182)	(0.143)
Distance to public transport			0.001	0.000
			(0.003)	(0.002)
Log pre-intervention CO_2_ emissions				$-0.047$
				(0.059)
Pre-intervention trips (total)				0.003
				(0.003)
Pre-intervention trips by car				$$-0.016^{**}$$
				(0.007)
Pre-intervention trips by public transport				$$0.138^{***}$$
				(0.012)
Pre-intervention trips by bike				0.008
				(0.013)
Constant	$$2.181^{***}$$	$$3.685^{***}$$	$$4.130^{***}$$	$$2.437^{***}$$
	(0.144)	(0.265)	(0.282)	(0.297)
Observations	820	820	820	820

The table presents estimates for the difference-in-differences model in Eq. (1), with the weekly distance of public transport trips as outcome variable. Column (1) depicts a regression of $log(Distance+1)$ on $Treatment, Time$, and $Treatment \times Time$. Column (2) adds participants’ age, gender language, above-median income, and higher education as additional control variables. Column (3) adds degree of urbanisation and distance to public transport (in km) as control variables. Column (4) further adds pre-intervention mobility behavior as control variables

The values in parentheses represent heteroskedasticity robust standard errors. *, **, ***, indicate statistical significance at the 10%, 5%, and 1% level

**Table 8:** Regression results (bike trips)

	$$log(Distance+1)$$			
	(1)	(2)	(3)	(4)
Time	0.031	0.031	0.031	0.031
	(0.135)	(0.134)	(0.134)	(0.081)
Treatment	0.072	0.072	0.066	0.007
	(0.144)	(0.144)	(0.144)	(0.081)
Treatment × Time	0.017	0.017	0.017	0.017
	(0.188)	(0.187)	(0.187)	(0.114)
Age		0.004	0.004	0.002
		(0.003)	(0.003)	(0.002)
Female (reference: male)		$$-0.179^{*}$$	$$-0.186^{**}$$	$$-0.172^{***}$$
		(0.094)	(0.094)	(0.060)
Monthly income CHF 9000 and higher		0.026	0.039	$$0.138^{**}$$
		(0.095)	(0.095)	(0.057)
Higher education		0.089	0.100	$-0.065$
		(0.112)	(0.112)	(0.070)
French (reference: German)		$$-0.252^{**}$$	$$-0.241^{**}$$	$$-0.117^{*}$$
		(0.103)	(0.104)	(0.067)
Italian (reference: German)		$-0.230$	$-0.233$	$-0.080$
		(0.190)	(0.190)	(0.114)
Medium density zip code (reference: high)			$-0.120$	$-0.016$
			(0.109)	(0.072)
Low density zip code (reference: high)			$-0.243^{*}$	$-0.022$
			(0.131)	(0.086)
Distance to public transport			$$-0.002^{**}$$	$$-0.000$$
			(0.001)	(0.000)
Log pre-intervention CO_2_ emissions				$$0.080^{**}$$
				(0.031)
Pre-intervention trips (total)				$0.004^{*}$
				(0.002)
Pre-intervention trips by car				$-0.007^{*}$
				(0.004)
Pre-intervention trips by public transport				$-0.007$
				(0.006)
Pre-intervention trips by bike				$$0.228^{***}$$
				(0.009)
Constant	$$1.080^{***}$$	$$1.028^{***}$$	$$1.187^{***}$$	0.208
Observations	(0.105)	(0.180)	(0.195)	(0.150)
	820	820	820	820

The table presents estimates for the difference-in-differences model in Eq. (1), with the weekly distance of bike trips as outcome variable. Column (1) depicts a regression of $log(Distance+1)$ on $Treatment, Time$, and $Treatment \times Time$. Column (2) adds participants’ age, gender language, above-median income, and higher education as additional control variables. Column (3) adds degree of urbanisation and distance to public transport (in km) as control variables. Column (4) further adds pre-intervention mobility behavior as control variables

The values in parentheses represent heteroskedasticity robust standard errors. *, **, ***, indicate statistical significance at the 10%, 5%, and 1% level

**Table 9:** Regression results (walk trips)

	$$log(Distance+1)$$			
	(1)	(2)	(3)	(4)
Time	$-0.061$	$-0.061$	$-0.061$	$-0.061$
	(0.082)	(0.082)	(0.081)	(0.061)
Treatment	$-0.013$	$-0.015$	$-0.026$	$-0.014$
	(0.087)	(0.087)	(0.086)	(0.062)
Treatment × Time	0.097	0.097	0.097	0.097
	(0.112)	(0.112)	(0.110)	(0.082)
Age		$-0.002$	$-0.002$	0.001
		(0.002)	(0.002)	(0.001)
Female (reference: male)		$$-0.146^{**}$$	$$-0.151^{***}$$	$$-0.024$$
		(0.059)	(0.058)	(0.044)
Monthly income CHF 9000 and higher		$-0.006$	0.012	0.065
		(0.057)	(0.055)	(0.042)
Higher education		$-0.116^{*}$	$-0.097$	0.053
		(0.062)	(0.061)	(0.050)
French (reference: German)		$-0.034$	$-0.021$	$-0.063$
		(0.066)	(0.065)	(0.048)
Italian (reference: German)		0.080	0.089	0.082
		(0.091)	(0.096)	(0.075)
Medium density zip code (reference: high)			$$-0.222^{***}$$	$$-0.036$$
			(0.064)	(0.049)
Low density zip code (reference: high)			$$-0.375^{***}$$	$$-0.105^{*}$$
			(0.076)	(0.062)
Distance to public transport			$$-0.002^{***}$$	$$-0.001^{**}$$
			(0.000)	(0.000)
Log pre-intervention CO_2_ emissions				$$-0.072^{***}$$
				(0.025)
Pre-intervention trips (total)				$$0.035^{***}$$
				(0.002)
Pre-intervention trips by car				$$-0.034^{***}$$
				(0.003)
Pre-intervention trips by public transport				$$-0.048^{***}$$
				(0.004)
Pre-intervention trips by bike				$$-0.028^{***}$$
				(0.005)
Constant	$$2.410^{***}$$	$$2.595^{***}$$	$$2.834^{***}$$	$$1.795^{***}$$
	(0.063)	(0.117)	(0.124)	(0.115)
Observations	820	820	820	820

The table presents estimates for the difference-in-differences model in Eq. (1), with the weekly distance of walk trips as outcome variable. Column (1) depicts a regression of $log(Distance+1)$ on $Treatment, Time$, and $Treatment \times Time$. Column (2) adds participants’ age, gender language, above-median income, and higher education as additional control variables. Column (3) adds degree of urbanisation and distance to public transport (in km) as control variables. Column (4) further adds pre-intervention mobility behavior as control variables

The values in parentheses represent heteroskedasticity robust standard errors. *, **, ***, indicate statistical significance at the 10%, 5%, and 1% level

**Table 10:** Regression results (low density sub-sample)

	$$log({CO_{2}}+1)$$			
	(1)	(2)	(3)	(4)
Time	$-0.043$	$-0.043$	$-0.043$	$-0.043$
	(0.203)	(0.202)	(0.201)	(0.090)
Treatment	$-0.148$	$-0.114$	$-0.146$	$-0.002$
	(0.208)	(0.209)	(0.209)	(0.052)
Treatment × Time	0.238	0.238	0.238	$0.238^{*}$
	(0.273)	(0.267)	(0.263)	(0.127)
Age		0.001	0.003	0.001
		(0.004)	(0.004)	(0.002)
Female (reference: male)		$$-0.338^{**}$$	$$-0.337^{**}$$	$$-0.024$$
		(0.153)	(0.155)	(0.089)
Monthly income CHF 9000 and higher		$-0.190$	$-0.264^{*}$	$-0.019$
		(0.149)	(0.151)	(0.069)
Higher education		0.157	0.099	0.092
		(0.141)	(0.135)	(0.069)
French (reference: German)		$$0.267^{*}$$	$$0.296^{**}$$	0.026
		(0.137)	(0.133)	(0.068)
Italian (reference: German)		$$0.969^{***}$$	$$0.930^{***}$$	$$-0.109$$
		(0.300)	(0.320)	(0.253)
Distance to public transport			$$0.010^{**}$$	0.003
			(0.005)	(0.003)
Log pre-intervention CO_2_ emissions				$$0.731^{***}$$
				(0.090)
Pre-intervention trips (total)				$-0.000$
				(0.002)
Pre-intervention trips by car				$0.009^{*}$
				(0.005)
Pre-intervention trips by public transport				$-0.002$
				(0.005)
Pre-intervention trips by bike				0.009
				(0.009)
Constant	$$3.777^{***}$$	$$3.793^{***}$$	$$3.608^{***}$$	$$0.747^{**}$$
	(0.144)	(0.328)	(0.343)	(0.341)
Observations	182	182	182	182

The table presents estimates for the difference-in-differences model in Eq. (1), using only participants in low density zip codes. Column (1) depicts a regression of $log({CO_{2}}+1)$ on $Treatment, Time$, and $Treatment \times Time$. Column (2) adds participants’ age, gender language, above-median income, and higher education as additional control variables. Column (3) adds degree of urbanisation and distance to public transport (in km) as control variables. Column (4) further adds pre-intervention mobility behavior as control variables

The values in parentheses represent heteroskedasticity robust standard errors. *, **, ***, $*{*}*$ indicate statistical significance at the 10%, 5%, and 1% level

**Table 11:** Regression results (medium density sub-sample)

	$$log({CO_{2}}+1)$$			
	(1)	(2)	(3)	(4)
Time	$$0.302^{**}$$	$$0.302^{**}$$	$$0.302^{**}$$	$$0.302^{***}$$
	(0.153)	(0.152)	(0.152)	(0.081)
Treatment	$0.301^{*}$	$0.310^{*}$	$0.310^{*}$	$0.077^{*}$
	(0.159)	(0.159)	(0.160)	(0.041)
Treatment × Time	$-0.180$	$-0.180$	$-0.180$	$-0.180^{*}$
	(0.211)	(0.211)	(0.211)	(0.106)
Age		$-0.003$	$-0.004$	$-0.004^{*}$
		(0.004)	(0.004)	(0.002)
Female (reference: male)		$-0.036$	$-0.042$	$-0.066$
		(0.112)	(0.112)	(0.052)
Monthly income CHF 9000 and higher		0.056	0.054	0.072
		(0.104)	(0.104)	(0.051)
Higher education		$$0.217^{**}$$	$$0.217^{**}$$	$$-0.006$$
		(0.101)	(0.102)	(0.057)
French (reference: German)		$-0.174$	$-0.165$	$-0.069$
		(0.133)	(0.131)	(0.056)
Italian (reference: German)		$-0.271$	$-0.273$	$-0.026$
		(0.207)	(0.208)	(0.097)
Distance to public transport			$-0.001$	$-0.002$
			(0.002)	(0.001)
Log pre-intervention CO_2_ emissions				$$0.750^{***}$$
				(0.053)
Pre-intervention trips (total)				$-0.004^{*}$
				(0.002)
Pre-intervention trips by car				$$0.012^{**}$$
				(0.005)
Pre-intervention trips by public transport				$0.010^{*}$
				(0.006)
Pre-intervention trips by bike				0.007
				(0.007)
Constant	$$3.206^{***}$$	$$3.350^{***}$$	$$3.382^{***}$$	$$0.934^{***}$$
	(0.117)	(0.252)	(0.259)	(0.197)
Observations	394	394	394	394

The table presents estimates for the difference-in-differences model in Eq. (1), using only participants in medium density zip codes. Column (1) depicts a regression of $log({CO_{2}}+1)$ on $Treatment, Time$, and $Treatment \times Time$. Column (2) adds participants’ age, gender language, above-median income, and higher education as additional control variables. Column (3) adds degree of urbanisation and distance to public transport (in km) as control variables. Column (4) further adds pre-intervention mobility behavior as control variables

The values in parentheses represent heteroskedasticity robust standard errors. *, **, *** indicate statistical significance at the 10%, 5%, and 1% level

**Table 12:** Regression results (high density sub-sample)

	$$log({CO_{2}}+1)$$			
	(1)	(2)	(3)	(4)
Time	0.374	0.374	0.374	$$0.374^{***}$$
	(0.243)	(0.229)	(0.229)	(0.142)
Treatment	0.281	0.220	0.235	0.031
	(0.250)	(0.240)	(0.240)	(0.055)
Treatment × Time	$-0.238$	$-0.238$	$-0.238$	$-0.238$
	(0.333)	(0.318)	(0.317)	(0.169)
Age		$-0.009^{*}$	$-0.009^{*}$	0.001
		(0.005)	(0.005)	(0.003)
Female (reference: male)		$$-0.642^{***}$$	$$-0.593^{***}$$	$$-0.038$$
		(0.164)	(0.162)	(0.085)
Monthly income CHF 9000 and higher		0.087	0.010	0.043
		(0.167)	(0.173)	(0.085)
Higher education		$0.339^{*}$	$0.365^{*}$	0.099
		(0.197)	(0.197)	(0.096)
French (reference: German)		$$-0.451^{**}$$	$$-0.411^{**}$$	$$-0.016$$
		(0.194)	(0.198)	(0.109)
Italian (reference: German)		$$0.381^{*}$$	$$0.441^{**}$$	$$-0.044$$
		(0.207)	(0.212)	(0.128)
Distance to public transport			$$0.007^{**}$$	0.003
			(0.003)	(0.003)
Log pre-intervention CO_2_ emissions				$$0.786^{***}$$
				(0.055)
Pre-intervention trips (total)				0.003
				(0.003)
Pre-intervention trips by car				0.003
				(0.006)
Pre-intervention trips by public transport				$-0.006$
				(0.008)
Pre-intervention trips by bike				$-0.002$
				(0.010)
Constant	$$2.492^{***}$$	$$3.219^{***}$$	$$3.107^{***}$$	0.312
	(0.184)	(0.300)	(0.299)	(0.198)
Observations	244	244	244	244

The table presents estimates for the difference-in-differences model in Eq. (1), using only participants in high density zip codes. Column (1) depicts a regression of $log({CO_{2}}+1)$ on $Treatment, Time$, and $Treatment \times Time$. Column (2) adds participants’ age, gender language, above-median income, and higher education as additional control variables. Column (3) adds degree of urbanisation and distance to public transport (in km) as control variables. Column (4) further adds pre-intervention mobility behavior as control variables

The values in parentheses represent heteroskedasticity robust standard errors. *, **, ***, indicate statistical significance at the 10%, 5%, and 1% level

**Table 13:** Regression results (low emission sub-sample)

	$$log({CO_{2}}+1)$$			
	(1)	(2)	(3)	(4)
Time	$$0.592^{***}$$	$$0.592^{***}$$	$$0.592^{***}$$	$$0.592^{***}$$
	(0.139)	(0.134)	(0.131)	(0.094)
Treatment	0.118	0.154	0.185	0.034
	(0.139)	(0.138)	(0.133)	(0.035)
Treatment × Time	$-0.161$	$-0.161$	$-0.161$	$-0.161$
	(0.200)	(0.196)	(0.191)	(0.120)
Age		$-0.003$	$-0.002$	$-0.001$
		(0.003)	(0.003)	(0.002)
Female (reference: male)		$$-0.305^{***}$$	$$-0.297^{***}$$	$$-0.042$$
		(0.100)	(0.096)	(0.064)
Monthly income CHF 9000 and higher		$$0.209^{**}$$	$$0.180^{*}$$	0.040
		(0.101)	(0.098)	(0.061)
Higher education		$0.193^{*}$	0.160	0.059
		(0.112)	(0.107)	(0.068)
French (reference: German)		$$-0.252^{**}$$	$$-0.221^{**}$$	$$-0.071$$
		(0.114)	(0.110)	(0.068)
Italian (reference: German)		0.027	$-0.003$	$-0.095$
		(0.169)	(0.185)	(0.104)
Medium density zip code (reference: high)			$$0.440^{***}$$	$$0.133^{*}$$
			(0.107)	(0.070)
Low density zip code (reference: high)			$$0.568^{***}$$	0.091
			(0.134)	(0.094)
Distance to public transport			$-0.000$	$-0.001$
			(0.002)	(0.002)
Log pre-intervention CO_2_ emissions				$$0.757^{***}$$
				(0.055)
Pre-intervention trips (total)				$-0.000$
				(0.002)
Pre-intervention trips by car				0.008
				(0.006)
Pre-intervention trips by public transport				0.000
				(0.007)
Pre-intervention trips by bike				0.002
				(0.007)
Constant	$$2.213^{***}$$	$$2.391^{***}$$	$$2.100^{***}$$	$$0.500^{***}$$
	(0.096)	(0.184)	(0.180)	(0.159)
Observations	410	410	410	410

$^1$ The table presents estimates for the difference-in-differences model in Eq. (1), using only participants with below median pre-intervention emissions. Column (1) depicts a regression of $log({CO_{2}}+1)$ on $Treatment, Time$, and $Treatment \times Time$. Column (2) adds participants’ age, gender language, above-median income, and higher education as additional control variables. Column (3) adds degree of urbanisation and distance to public transport (in km) as control variables. Column (4) further adds pre-intervention mobility behavior as control variables

The values in parentheses represent heteroskedasticity robust standard errors. *, **, ***, indicate statistical significance at the 10%, 5%, and 1% level

**Table 14:** Regression results (high emission sub-sample)

	$$log({CO_{2}}+1)$$			
	(1)	(2)	(3)	(4)
Time	$$-0.136$$	$$-0.136$$	$$-0.136$$	$$-0.136^{**}$$
	(0.091)	(0.087)	(0.086)	(0.063)
Treatment	0.010	$-0.017$	$-0.016$	0.008
	(0.076)	(0.074)	(0.074)	(0.019)
Treatment × Time	0.017	0.017	0.017	0.017
	(0.125)	(0.122)	(0.121)	(0.083)
Age		$$-0.006^{***}$$	$$-0.006^{***}$$	$$-0.002$$
		(0.002)	(0.002)	(0.002)
Female (reference: male)		$$-0.265^{***}$$	$$-0.270^{***}$$	$$-0.048$$
		(0.068)	(0.070)	(0.051)
Monthly income CHF 9000 and higher		$-0.075$	$-0.098$	0.050
		(0.061)	(0.063)	(0.044)
Higher education		0.055	0.053	0.005
		(0.064)	(0.064)	(0.042)
French (reference: German)		0.065	0.059	$-0.003$
		(0.073)	(0.074)	(0.046)
Italian (reference: German)		0.182	0.159	$-0.021$
		(0.135)	(0.134)	(0.101)
Medium density zip code (reference: high)			0.129	0.078
			(0.095)	(0.065)
Low density zip code (reference: high)			$$0.219^{**}$$	0.080
			(0.093)	(0.065)
Distance to public transport			$0.002^{*}$	0.001
			(0.001)	(0.001)
Lg pre-intervention CO_2_ emissions				$$0.761^{***}$$
				(0.068)
Pre-intervention trips (total)				$-0.002$
				(0.002)
Pre-intervention trips by car				$$0.009^{**}$$
				(0.004)
Pre-intervention trips by public transport				0.004
				(0.004)
Pre-intervention trips by bike				0.004
				(0.007)
Constant	$$4.163^{***}$$	$$4.576^{***}$$	$$4.421^{***}$$	$$0.890^{***}$$
	(0.055)	(0.143)	(0.155)	(0.292)
Observations	410	410	410	410

The table presents estimates for the difference-in-differences model in Eq. (1), using only participants with above median pre-intervention emissions. Column (1) depicts a regression of $log({CO_{2}}+1)$ on $Treatment, Time$, and $Treatment \times Time$. Column (2) adds participants’ age, gender language, above-median income, and higher education as additional control variables. Column (3) adds degree of urbanisation and distance to public transport (in km) as control variables. Column (4) further adds pre-intervention mobility behavior as control variables

The values in parentheses represent heteroskedasticity robust standard errors. *, **, ***, indicate statistical significance at the 10%, 5%, and 1% level

**Table 15:** Regression results (young sub-sample)

	$$log({CO_{2}}+1)$$			
	(1)	(2)	(3)	(4)
Time	0.220	0.220	0.220	$$0.220^{**}$$
	(0.178)	(0.178)	(0.172)	(0.090)
Treatment	0.127	0.137	0.169	0.035
	(0.180)	(0.181)	(0.178)	(0.040)
Treatment × Time	$-0.058$	$-0.058$	$-0.058$	$-0.058$
	(0.241)	(0.240)	(0.233)	(0.114)
Age		$-0.010$	$-0.005$	$-0.003$
		(0.008)	(0.007)	(0.004)
Female (reference: male)		$-0.057$	$-0.160$	$-0.040$
		(0.128)	(0.128)	(0.060)
Monthly income CHF 9000 and higher		0.060	0.035	0.090
		(0.121)	(0.118)	(0.058)
Higher education		$$0.280^{**}$$	$$0.242^{*}$$	0.019
		(0.132)	(0.131)	(0.067)
French (reference: German)		$-0.214$	$-0.217$	$-0.096$
		(0.151)	(0.145)	(0.059)
Italian (reference: German)		$-0.363$	$-0.455$	0.036
		(0.301)	(0.292)	(0.147)
Medium density zip code (reference: high)			$$0.621^{***}$$	$$0.134^{*}$$
			(0.144)	(0.077)
Low density zip code (reference: high)			$$0.802^{***}$$	$$0.127^{*}$$
			(0.154)	(0.075)
Distance to public transport			$-0.001$	$-0.001$
			(0.002)	(0.001)
Log pre-intervention CO_2_ emissions				$$0.778^{***}$$
				(0.046)
Pre-intervention trips (total)				$-0.002$
				(0.002)
Pre-intervention trips by car				$0.009^{*}$
				(0.005)
Pre-intervention trips by public transport				0.005
				(0.006)
Pre-intervention trips by bike				$-0.006$
				(0.009)
Constant	$$3.171^{***}$$	$$3.492^{***}$$	$$2.952^{***}$$	$$0.732^{***}$$
	(0.133)	(0.275)	(0.272)	(0.181)
Observations	420	420	420	420

The table presents estimates for the difference-in-differences model in Eq. (1), using only participants of below median age. Column (1) depicts a regression of $log({CO_{2}}+1)$ on $Treatment, Time$, and $Treatment \times Time$. Column (2) adds participants’ age, gender language, above-median income, and higher education as additional control variables. Column (3) adds degree of urbanisation and distance to public transport (in km) as control variables. Column (4) further adds pre-intervention mobility behavior as control variables

The values in parentheses represent heteroskedasticity robust standard errors $$*$$, $$**$$, $$*{*}*$$ indicate statistical significance at the 10%, 5%, and 1% level

**Table 16:** Regression results (old sub-sample)

	$$log({CO_{2}}+1)$$			
	(1)	(2)	(3)	(4)
Time	$$0.274^{*}$$	$$0.274^{*}$$	$$0.274^{**}$$	$$0.274^{***}$$
	(0.153)	(0.148)	(0.135)	(0.079)
Treatment	0.188	0.169	0.208	0.039
	(0.167)	(0.164)	(0.148)	(0.035)
Treatment × Time	$-0.150$	$-0.150$	$-0.150$	$-0.150$
	(0.219)	(0.212)	(0.195)	(0.101)
Age		$-0.008$	$-0.005$	$-0.002$
		(0.006)	(0.006)	(0.004)
Female (reference: male)		$$-0.466^{***}$$	$$-0.355^{***}$$	$$-0.071$$
		(0.117)	(0.106)	(0.057)
Monthly income CHF 9000 and higher		0.055	$-0.024$	$-0.005$
		(0.111)	(0.104)	(0.056)
Higher education		$$0.398^{***}$$	$$0.300^{***}$$	0.009
		(0.118)	(0.109)	(0.052)
French (reference: German)		$-0.038$	$-0.024$	0.021
		(0.122)	(0.116)	(0.066)
Italian (reference: German)		0.212	0.181	$-0.094$
		(0.207)	(0.195)	(0.091)
Medium density zip code (reference: high)			$$0.757^{***}$$	0.041
			(0.136)	(0.069)
Low density zip code (reference: high)			$$1.159^{***}$$	0.039
			(0.148)	(0.083)
Distance to public transport			$$0.006^{***}$$	0.001
			(0.002)	(0.001)
Log pre-intervention CO_2_ emissions				$$0.762^{***}$$
				(0.044)
Pre-intervention trips (total)				0.000
				(0.002)
Pre-intervention trips by car				$$0.007^{**}$$
				(0.003)
Pre-intervention trips by public transport				$-0.002$
				(0.005)
Pre-intervention trips by bike				$0.010^{*}$
				(0.006)
Constant	$$3.105^{***}$$	$$3.609^{***}$$	$$2.736^{***}$$	$$0.700^{**}$$
	(0.119)	(0.424)	(0.436)	(0.291)
Observations	400	400	400	400

The table presents estimates for the difference-in-differences model in Eq. (1), using only participants of above median age. Column (1) depicts a regression of $log({CO_{2}}+1)$ on $Treatment, Time$, and $Treatment \times Time$. Column (2) adds participants’ age, gender language, above-median income, and higher education as additional control variables. Column (3) adds degree of urbanisation and distance to public transport (in km) as control variables. Column (4) further adds pre-intervention mobility behavior as control variables

The values in parentheses represent heteroskedasticity robust standard errors. *, **, ***, indicate statistical significance at the 10%, 5%, and 1% level

**Table 17:** Regression results (male sub-sample)

	$$log({CO_{2}}+1)$$			
	(1)	(2)	(3)	(4)
Time	$$0.292^{*}$$	$$0.292^{*}$$	$$0.292^{*}$$	$$0.292^{***}$$
	(0.154)	(0.153)	(0.150)	(0.084)
Treatment	0.238	0.252	$0.279^{*}$	0.051
	(0.165)	(0.165)	(0.158)	(0.036)
Treatment × Time	$$-0.211$$	$$-0.211$$	$$-0.211$$	$$-0.211^{**}$$
	(0.222)	(0.220)	(0.213)	(0.107)
Age		0.002	0.001	$-0.000$
		(0.003)	(0.003)	(0.002)
Monthly income CHF 9000 and higher		$$0.209^{*}$$	0.108	$$0.114^{**}$$
		(0.112)	(0.109)	(0.056)
Higher education		$0.216^{*}$	0.150	0.007
		(0.112)	(0.111)	(0.053)
French (reference: German)		$$-0.212^{*}$$	$$-0.273^{**}$$	$$-0.074$$
		(0.127)	(0.122)	(0.064)
Italian (reference: German)		$-0.148$	$-0.202$	$-0.149$
		(0.247)	(0.235)	(0.096)
Medium density zip code (reference: high)			$$0.431^{***}$$	0.081
			(0.136)	(0.071)
Low density zip code (reference: high)			$$0.873^{***}$$	0.036
			(0.143)	(0.082)
Distance to public transport			$-0.000$	$-0.001$
			(0.002)	(0.001)
Log pre-intervention CO_2_ emissions				$$0.776^{***}$$
				(0.049)
Pre-intervention trips (total)				$-0.001$
				(0.002)
Pre-intervention trips by car				$$0.007^{**}$$
				(0.003)
Pre-intervention trips by public transport				0.001
				(0.005)
Pre-intervention trips by bike				0.004
				(0.007)
Constant	$$3.211^{***}$$	$$3.045^{***}$$	$$2.783^{***}$$	$$0.607^{***}$$
	(0.119)	(0.221)	(0.225)	(0.176)
Observations	432	432	432	432

The table presents estimates for the difference-in-differences model in Eq. (1), using only male participants. Column (1) depicts a regression of $log({CO_{2}}+1)$ on $Treatment, Time$, and $Treatment \times Time$. Column (2) adds participants’ age, gender language, above-median income, and higher education as additional control variables. Column (3) adds degree of urbanisation and distance to public transport (in km) as control variables. Column (4) further adds pre-intervention mobility behavior as control variables

The values in parentheses represent heteroskedasticity robust standard errors. *, **, ***,  indicate statistical significance at the 10%, 5%, and 1% level

**Table 18:** Regression results (female sub-sample)

	$$log({CO_{2}}+1)$$			
	(1)	(2)	(3)	(4)
Time	0.192	0.192	0.192	$$0.192^{**}$$
	(0.180)	(0.177)	(0.155)	(0.086)
Treatment	0.084	0.044	0.090	0.019
	(0.182)	(0.181)	(0.168)	(0.039)
Treatment × Time	0.016	0.016	0.016	0.016
	(0.239)	(0.235)	(0.215)	(0.109)
Age		$$-0.014^{***}$$	$$-0.011^{***}$$	$$-0.003$$
		(0.004)	(0.004)	(0.002)
Monthly income CHF 9000 and higher		$-0.076$	$-0.045$	$-0.016$
		(0.118)	(0.111)	(0.055)
Higher education		$$0.444^{***}$$	$$0.376^{***}$$	0.051
		(0.127)	(0.117)	(0.068)
French (reference: German)		$-0.024$	0.063	0.011
		(0.151)	(0.130)	(0.062)
Italian (reference: German)		0.094	0.118	0.025
		(0.232)	(0.232)	(0.114)
Medium density zip code (reference: high)			$$0.949^{***}$$	$$0.133^{*}$$
			(0.138)	(0.075)
Low density zip code (reference: high)			$$1.050^{***}$$	$$0.136^{*}$$
			(0.163)	(0.081)
Distance to public transport			$$0.005^{**}$$	0.002
			(0.003)	(0.001)
Log pre-intervention CO_2_ emissions				$$0.729^{***}$$
				(0.044)
Pre-intervention trips (total)				$-0.002$
				(0.003)
Pre-intervention trips by car				$$0.013^{**}$$
				(0.006)
Pre-intervention trips by public transport				0.007
				(0.006)
Pre-intervention trips by bike				0.004
				(0.008)
Constant	$$3.052^{***}$$	$$3.580^{***}$$	$$2.618^{***}$$	$$0.704^{***}$$
	(0.136)	(0.227)	(0.230)	(0.175)
Observations	388	388	388	388

The table presents estimates for the difference-in-differences model in Eq. (1), using only female participants. Column (1) depicts a regression of $log({CO_{2}}+1)$ on $Treatment, Time$, and $Treatment \times Time$. Column (2) adds participants’ age, gender language, above-median income, and higher education as additional control variables. Column (3) adds degree of urbanisation and distance to public transport (in km) as control variables. Column (4) further adds pre-intervention mobility behavior as control variables

The values in parentheses represent heteroskedasticity robust standard errors. *, **, ***, indicate statistical significance at the 10%, 5%, and 1% level

**Table 19:** Regression results (sub-sample without screen events)

	$$log({CO_{2}}+1)$$			
	(1)	(2)	(3)	(4)
Time	$$0.247^{**}$$	$$0.247^{**}$$	$$0.247^{**}$$	$$0.247^{***}$$
	(0.118)	(0.117)	(0.111)	(0.060)
Treatment	0.297	$$0.349^{*}$$	$$0.421^{**}$$	$$0.075^{*}$$
	(0.207)	(0.205)	(0.194)	(0.045)
Treatment × Time	$-0.086$	$-0.086$	$-0.086$	$-0.086$
	(0.278)	(0.275)	(0.258)	(0.115)
Age		$-0.004$	$-0.005$	$-0.002$
		(0.003)	(0.003)	(0.002)
Female (reference: male)		$$-0.193^{*}$$	$$-0.246^{**}$$	$$-0.092^{*}$$
		(0.110)	(0.108)	(0.053)
Monthly income CHF 9000 and higher		$0.181^{*}$	0.089	0.083
		(0.104)	(0.102)	(0.054)
Higher education		$$0.344^{***}$$	$$0.253^{**}$$	0.012
		(0.111)	(0.109)	(0.055)
French (reference: German)		$-0.015$	$-0.013$	0.008
		(0.120)	(0.113)	(0.057)
Italian (reference: German)		0.130	0.096	0.045
		(0.246)	(0.238)	(0.116)
Medium density zip code (reference: high)			$$0.731^{***}$$	0.088
			(0.130)	(0.075)
Low density zip code (reference: high)			$$1.036^{***}$$	0.001
			(0.147)	(0.082)
Distance to public transport			$-0.001$	$-0.002$
			(0.002)	(0.002)
Log pre-intervention CO_2_ emissions				$$0.766^{***}$$
				(0.044)
Pre-intervention trips (total)				$-0.003$
				(0.002)
Pre-intervention trips by car				$$0.009^{**}$$
				(0.004)
Pre-intervention trips by public transport				0.002
				(0.006)
Pre-intervention trips by bike				0.007
				(0.007)
Constant	$$3.139^{***}$$	$$3.237^{***}$$	$$2.756^{***}$$	$$0.786^{***}$$
	(0.090)	(0.206)	(0.205)	(0.161)
Observations	488	488	488	488

$^1$ The table presents estimates for the difference-in-differences model in Eq. (1), using only those treatment group participants who did not interact with the SCC App. Column (1) depicts a regression of $log({CO_{2}}+1)$ on $Treatment, Time$, and $Treatment \times Time$. Column (2) adds participants’ age, gender language, above-median income, and higher education as additional control variables. Column (3) adds degree of urbanisation and distance to public transport (in km) as control variables. Column (4) further adds pre-intervention mobility behavior as control variables

The values in parentheses represent heteroskedasticity robust standard errors. *, **, ***,   indicate statistical significance at the 10%, 5%, and 1% level

**Table 20:** Regression results (sub-sample with screen events)

	$$log({CO_{2}}+1)$$			
	(1)	(2)	(3)	(4)
Time	$$0.247^{**}$$	$$0.247^{**}$$	$$0.247^{**}$$	$$0.247^{***}$$
	(0.118)	(0.116)	(0.111)	(0.060)
Treatment	0.118	0.113	0.138	0.035
	(0.130)	(0.129)	(0.122)	(0.027)
Treatment × Time	$-0.108$	$-0.108$	$-0.108$	$-0.108$
	(0.172)	(0.169)	(0.161)	(0.081)
Age		$-0.005^{*}$	$-0.005^{*}$	$-0.002$
		(0.003)	(0.003)	(0.001)
Female (reference: male)		$$-0.284^{***}$$	$$-0.289^{***}$$	$$-0.056$$
		(0.089)	(0.084)	(0.046)
Monthly income CHF 9000 and higher		0.042	0.022	0.061
		(0.086)	(0.082)	(0.042)
Higher education		$$0.269^{***}$$	$$0.228^{***}$$	0.051
		(0.089)	(0.085)	(0.043)
French (reference: German)		$$-0.224^{**}$$	$$-0.216^{**}$$	$$-0.041$$
		(0.101)	(0.094)	(0.049)
Italian (reference: German)		$-0.155$	$-0.186$	$-0.098$
		(0.172)	(0.168)	(0.077)
Medium density zip code (reference: high)			$$0.651^{***}$$	$$0.103^{*}$$
			(0.102)	(0.055)
Low density zip code (reference: high)			$$0.961^{***}$$	0.098
			(0.112)	(0.060)
Distance to public transport			$-0.000$	$-0.001$
			(0.002)	(0.001)
Log pre-intervention CO_2_ emissions				$$0.759^{***}$$
				(0.034)
Pre-intervention trips (total)				$-0.002$
				(0.002)
Pre-intervention trips by car				$$0.008^{**}$$
				(0.003)
Pre-intervention trips by public transport				0.003
				(0.004)
Pre-intervention trips by bike				0.002
				(0.005)
Constant	$$3.139^{***}$$	$$3.462^{***}$$	$$2.944^{***}$$	$$0.699^{***}$$
	(0.089)	(0.182)	(0.181)	(0.141)
Observations	728	728	728	728

The table presents estimates for the difference-in-differences model in Eq. (1), using only those treatment group participants who did interact with the SCC App. Column (1) depicts a regression of $log({CO_{2}}+1)$ on $Treatment, Time$, and $Treatment \times Time$. Column (2) adds participants’ age, gender language, above-median income, and higher education as additional control variables. Column (3) adds degree of urbanisation and distance to public transport (in km) as control variables. Column (4) further adds pre-intervention mobility behavior as control variables

The values in parentheses represent heteroskedasticity robust standard errors. *, **, ***, indicate statistical significance at the 10%, 5%, and 1% level

## Spillover analysis

At the beginning and end of the study, participants were asked to answer a series of questions related to their other environmental behaviors. Participants reported eight environmental behaviors as displayed in Table 21. The response options ranged from of 1 (Never) to 7 (Always) on a Likert scale. Additionally, participants could respond with “I don’t know” or “I don’t want to specify” (in which case we impute the mode of the respective question).

We calculate the mean of the eight responses at the beginning and end of the study. Our outcome variable is the difference between these two values. Positive values in $\Delta SpilloverScore$ indicate a change to more environmentally friendly self reported behavior.

$$\begin{aligned} \Delta SpilloverScore = SpilloverScore_{end} - SpilloverScore_{beginning} \end{aligned}$$

Table 22 presents the results of our spillover analysis. Column (1) depicts a simple regression of $\Delta SpilloverScore$ on *Treatment*. Column (2) adds participants’ age, gender language, above-median income, and higher education as additional control variables. Column (3) adds degree of urbanisation and distance to public transport (in km) as control variables. Column (4) further adds pre-intervention mobility behavior as control variables. All three specifications find positive coefficients that are small and statistically insignificant.

**Table 21:** Survey on environmental behaviors

Environmental behaviors:	
This question asks you to describe your specific environmental behavior in more detail.	
In the past month, .	
I recycled paper, aluminum, glass and kitchen waste.	
I used public transportation or the bike instead of driving.	
I adjusted my clothing instead of turning on heaters/coolers to save energy.	
I did not throw away food.	
I bought seasonal, local or organic food.	
I replaced electrical appliances only when they were damaged.	
I have saved electricity.	
I have conserved water in personal routines (e.g., showering) and in doing household chores.

**Table 22:** Regression results (Spillover behaviors)

	Change in spillover behaviors			
	(1)	(2)	(3)	(4)
Treatment	0.074	0.050	0.050	0.049
	(0.067)	(0.067)	(0.067)	(0.068)
Age		$-0.001$	$-0.001$	$-0.001$
		(0.002)	(0.002)	(0.002)
Female (reference: male)		0.035	0.035	0.048
		(0.070)	(0.071)	(0.074)
Monthly income CHF 9000 and higher		$-0.110$	$-0.117^{*}$	$-0.109$
		(0.067)	(0.066)	(0.066)
Higher education		0.056	0.052	0.064
		(0.074)	(0.074)	(0.077)
French (reference: German)		$-0.121$	$-0.124$	$-0.116$
		(0.078)	(0.078)	(0.077)
Italian (reference: German)		0.235	0.240	$0.246^{*}$
		(0.152)	(0.150)	(0.142)
Medium density zip code (reference: high)			0.059	0.062
			(0.073)	(0.078)
Low density zip code (reference: high)			0.149	0.138
			(0.101)	(0.107)
Distance to public transport			0.000	0.000
			(0.001)	(0.001)
Log pre-intervention CO_2_ emissions				$-0.004$
				(0.040)
Pre-intervention trips (total)				$-0.001$
				(0.003)
Pre-intervention trips by car				0.004
				(0.006)
Pre-intervention trips by public transport				0.008
				(0.007)
Pre-intervention trips by bike				$-0.001$
				(0.007)
Constant	$-0.004$	0.087	0.020	$-0.044$
	(0.049)	(0.134)	(0.143)	(0.207)
Observations	378	378	378	378

The table presents estimates for the change in potential spillover behaviors. The spillover behaviors are expressed as a score that is based on the answer to a set of questions regarding other environmental behaviors. The questions were answered at the beginning and the end of the study. Column (1) depicts a regression of $\Delta SpilloverScore$ on $Treatment$. Column (2) adds participants’ age, gender language, above-median income, and higher education as additional control variables. Column (3) adds degree of urbanisation and distance to public transport (in km) as control variables. Column (4) further adds pre-intervention mobility behavior as control variables

The values in parentheses represent heteroskedasticity robust standard errors. *, **, ***, indicate statistical significance at the 10%, 5%, and 1% level

## Footnotes

1 Our experimental design does not allow to evaluate the different components of the SCC App separately. The sample size of our study does not provide adequate statistical power for such a detailed analysis.

2 See Appendix 1 for figures of the SCC App’s features: graphical feedback (Fig. 5), moral appeal (Fig. 6), and social comparison (Fig. 7).

3 At the time of the study, one Swiss Franc traded for approximately 1.10 US Dollars.

4 In Switzerland, COVID-19 restrictions were significant for most of 2021. At the beginning of the pre-intervention period, on April 19, restrictions were partly lifted. Restaurant teraces, museums and zoos were allowed to open, small events with up to 100 people became possible again, and in-person classes at universities (Bundesamt für Statistik, 2023) were allowed.

5 Appendix 2 provides English transcriptions of the intervention emails, which were originally sent to the participants in German, French, or Italian, according to their preferred language.

6 Tracking success is defined as granting the SCC App all required location tracking permissions, and having at most 72 h between any two trips (including “stay” trips, which are recorded when the phone is stationary). We acknowledge that this measure of tracking success is imperfect, as it does not account for malfunctioning spells shorter than 72 h (e.g., phones that run out of battery) and we cannot distinguish whether a phone is with its owner or somewhere else (e.g., left at home). At the beginning of the intervention, both groups included 285 participants. 198 participants in the control group and 212 participants in the treatment group successfully tracked their mobility behavior in the intervention period. This difference in attrition is not statistically significant.

7 Appendix 3 provides an overview of the study schedule.

8 Trips are detected and assigned to a mode of transport using the MotionTag algorithm. $\hbox {CO}_2$ emissions are calculated using “mobitool 2.0” emission factors: $197~\hbox {gCO}_2$/km for car, $7~\hbox {gCO}_2$/km for train, $37~\hbox {gCO}_2$/km for tram, and $145~\hbox {gCO}_2$/km for bus trips (see Table Z.1. in, Frischknecht and Tuchschmid, 2016). Walk and bike trips are not assigned a $\hbox {CO}_2$ value.

9 The corresponding *p*-values are obtained from a t-test for numerical variables, and from a chi square test for categorical variables.

10 The few observations with zero emissions appear sensible. 9 participants have zero emissions in the pre-intervention period, one of them also has no emissions in the intervention period. Most of them (6 out of 9) are located in high population density zip codes. Each of these 9 participants recorded several walk trips, 5 of them also used the bike. Alternative specifications (Poisson; emissions per km as the dependent variable; $arcsinh(CO_{2i,t})$ as the dependent variable; excluding participants with zero emissions, results not shown) yield essentially the same results.

11 We interpret coefficients using the standard formula $e^{0.247}-1 = 28\%$. Doing so with the outcome variable $log({{CO}_2}+1)$ rather than *log*(*CO*2) is acceptable when few observations have $CO_2$ values of zero (Wooldridge, 2012, p. 193).

12 Despite a comparably large sample size, we cannot rule out small effects on $\hbox {CO}_2$ emissions. Power calculations suggest that our study is able to detect a $\hbox {CO}_2$ reduction of $18\%$ (Cohen’s *d* 0.14, a small effect size according to Cohen, 2013) at the $5\%$ significance level with $80\%$ power. To detect, for instance, a reduction of $5\%$ (Cohen’s *d* 0.04), a sample size of 4, 700 participants completing the study would be required. This sample size was well beyond the scope and budget of our study.
